# Benefits of ellagic acid from grapes and pomegranates against colorectal cancer

**DOI:** 10.22088/cjim.8.3.226

**Published:** 2017

**Authors:** Sayedalireza Mirsane, Sayedmojtaba Mirsane

**Affiliations:** 1 School of Nursing and Midwifery, Kashan University of Medical Sciences, Kashan, Iran.; 2 Department of Education, Khansar, Isfahan, Iran.

**Keywords:** Grapes, pomegranates, colorectal cancer, gastric, ellagic acid

**Dear Editor,**

We read an interesting article entitled: “Cytokeratin7 expression in gastric and colorectal adenocarcinoma: Correlation with prognostic factors” in the Caspian Journal of Internal Medicine, 2015: 6 (4). We admire the authors of above paper for their valuable paper. In this article, it has been explained that in Iran, gastric and colorectal adenocarcinoma are the second and the fifth most common cancers respectively. It is then suggested to increase attention to prevention of colorectal cancer which is very important. On the other hand, almost all people consume fruits as the best and safest way to treatment and elimination of diseases. Fruits such as pomegranates and grapes have remedial purposes. It is worth noting that the foods that introduced in the Holy Qur'an as good and useful foodstuffs, have many beneficial effects on health human and even they have more effective spiritual effects on humans’ life. According to the Holy Qur'an, pomegranate and grape grow in the gardens of paradise (1-3).

Cancer develops when cells in the human body begin to grow out of control and crowd out normal cells (4). Certainly cancers are very dangerous, so attention to reduction and treatment is necessary especially colorectal cancer (CRC). Our goal is to evaluate the benefits of ellagic acid from pomegranates and grapes in CRC. Fruits and vegetables have been said to have a strong protective effect against CRC especially those with ellagic acid. The highest levels of ellagic acid are found in pomegranates and grapes. The anticarcinogenic effects of ellagic acid has been detected. Also, ellagic acid has an anti-inflammatory role in the treatment of chronic ulcerative colitis as to prevent the development of colon cancer (5-8). 

Scholars also suggested that ellagic acid is an efficient multiple-function protector against oxidative stress (9). Kao et al. have reported the anti-proliferative effects of ellagic acid on different CRC cell types (10). It should be noted that the pathway of PI3K/Akt plays a central role in tumor genesis in colon (11, 12) and ellagic acid can inhibit chemically-induced colorectal tumorigenesis via mechanism involving the inhibition of Akt phosphorylation at Ser473. Similarly, the inhibitory impact of ellagic acid on Akt phosphorylation (at Thr308 and Ser473) has been observed in another study (13, 14). Researchers have stated that ellagic acid and its metabolites can inhibit CaCo-2 (the Caco-2 cell line is a continuous cell of heterogeneous human epithelial colorectal adenocarcinoma cell) proliferation through cell cycle arrest (15). We conclude that grape and pomegranate juice consumption has remedial and preventive impacts in CRC. We recommend that further studies should be conducted to obtain the other benefits of ellagic acid and use the significant advantages in studies of intestinal health promoting properties and decrease rates of CRC.
